# Unexpected Serum and Urine Aluminum Concentrations in Pediatric Patients on Home Parenteral Nutrition

**DOI:** 10.3390/nu15163597

**Published:** 2023-08-17

**Authors:** Hanna Romanowska, Klaudia Bartoszewicz, Mikołaj Danko, Joanna Wielopolska, Katarzyna Popińska, Joanna Żydak, Marta Sibilska, Anna Borkowska, Agnieszka Szlagatys-Sidorkiewicz, Janusz Książyk

**Affiliations:** 1 Department of Pediatrics, Endocrinology, Diabetology, Metabolic Diseases and Cardiology of the Developmental Age, Pomeranian Medical University, Unii Lubelskiej 1, 71-252 Szczecin, Poland; klaudia.bartoszewicz@gmail.com (K.B.); joannakastrau89@gmail.com (J.W.); 2 Department of Pediatrics, Nutrition and Metabolic Diseases, The Children’s Memorial Health Institute, Al. Dzieci Polskich 20, 04-730 Warsaw, Poland; ikim@poczta.onet.pl (M.D.); kasia.popinska@wp.pl (K.P.); msibilska@poczta.onet.pl (M.S.); janusz@ksiazyk.pl (J.K.); 3 Department of Pediatrics, Gastroenterology, Allergology and Nutrition, Medical University of Gdansk, Nowe Ogrody 1-6, 80-803 Gdansk, Poland; anna.borkowska@gumed.edu.pl (A.B.); agnieszka.szlagatys-sidorkiewicz@gumed.edu.pl (A.S.-S.)

**Keywords:** aluminum, parenteral nutrition, newborns, children, blood serum, urine

## Abstract

The intravenous supply of aluminum (Al) present in parenteral nutrition solutions poses a high risk of the absorption of this element, which can result in metabolic bone disease, anemia, and neurological complications. The aim of this study is to determine the impact of long-term parenteral nutrition (PN) in children on serum Al concentration and its urinary excretion compared to healthy children. We evaluated serum Al concentrations and its urinary excretion in patients enrolled in the Polish home parenteral nutrition (HPN) program between 2004 and 2022. The study group included 83 patients and the control group consisted of 121 healthy children. In children whose PN was started in the neonatal period, we found higher serum Al concentrations and higher urinary Al excretion than in other subjects whose PN was started later. Only 12% of the children on chronic parenteral nutrition had serum Al concentrations of less than 5 μg/L. Healthy children in the control group had higher serum Al concentrations than those in the parenteral nutrition group, which may indicate the influence of one’s environment and diet on Al serum levels.

## 1. Introduction

The absorption of Al, a widely present element in the environment that plays no biological role [1], is restricted by natural protective barriers, primarily the gastrointestinal tract [2,3]. Any Al that enters the systemic circulation is primarily eliminated by the kidneys (approximately 95%) and also excreted through bile [1,3].

Patients who require long-term PN and have compromised gastrointestinal function are at risk of excessive Al accumulation from the PN solutions they receive. This excess Al can accumulate in various tissues such as the bones, liver, spleen, kidneys, parathyroid glands, and nervous system, posing potential health risks [3,4]. 

The excessive accumulation of Al can lead to toxic effects, including metabolic bone disease, anemia, and neurological complications [5,6,7,8,9]. Many complications associated with excessive Al exposure manifest after years of cumulative exposure [3,10,11]. 

The issue of Al presence in PN solutions has been discussed since the 1980s. Over the past three decades, efforts have been made to reduce Al contamination in the pharmaceutical formulations used to create PN mixtures. In 1986, the United States Food and Drug Administration (FDA) issued recommendations mandating the elimination of Al from PN formulations [5,12]. In 1991, the American Society of Clinical Nutrition (ASCN) and the American Society of Parenteral and Enteral Nutrition (ASPEN) Working Group on Standards for Aluminum Content of Parenteral Nutrition Solutions established safety thresholds for Al concentrations in patients on long-term PN, suggesting an intake of less than 2 mg/kg/day as a safe level [6,13].

In 2004, the FDA implemented a rule regulating Al concentrations of up to 25 μg/L in large-volume parenteral products (LVPs), which include water for injection, dextrose solutions, crystalline amino acids, and fat emulsions. However, no upper limit for Al contamination has been set for small-volume parenterals (SVPs), such as electrolytes and trace elements. Nevertheless, the labeling of Al content on SVPs has been made mandatory [4,5,14].

Currently, there are no specific European regulations controlling Al supply in PN. The European Food Safety Authority (EFSA) has established a tolerable weekly intake (TWI) of 1 mg Al/kg body weight for healthy individuals, while the World Health Organization (WHO) has set a provisional tolerable weekly intake (PTWI) of 2 mg Al/kg body weight. The relative exposure in children can be higher, reaching up to 2.3 mg/kg body weight/week [15,16]. The German Federal Environmental Agency has established provisional reference values for the general population, recommending urine concentrations below 15 μg/L and serum concentrations below 5 μg/L. The German Research Foundation suggests a reference value of 50 μg Al/g creatinine for the general population [16]. However, reference ranges for safe Al concentrations in serum and urine, specifically for children, are still unavailable.

Among the products used to prepare PN mixtures, calcium gluconate and phosphate formulations are the main contributors to high Al content [6,17]. Preterm infants, who have increased calcium and phosphate requirements, face an additional burden of Al toxicity risk in this patient group [5,11,18]. However, a significant reduction in Al supply has been achieved through changes in packaging and the replacement of casein hydrolysate with crystalline amino acids [5,6,18,19,20,21,22]. Despite these modifications, patients are still exposed to Al, mainly through SVPs.

Most of the existing literature focuses on measuring Al concentrations in PN mixtures [5,11,18,20,23,24]. There are relatively few studies that assess Al concentrations in the blood and urine of pediatric patients, especially those on long-term PN [4,12,18,24,25]. Some publications have investigated Al concentration and exposure concerning distant complications in patients receiving short-term PN (less than 30 days) [9,10,26,27,28].

The aim of this study is to determine the impact of long-term parenteral nutrition (PN) in children on serum Al concentration and its urinary excretion compared to healthy children. In order to solve the research problem, we collected samples from 83 patients on long-term total parenteral nutrition (TPN). The present study is the largest of its kind that we are aware of. We assessed serum Al levels in these patients and measured the urinary excretion of Al in 74 of them. Additionally, we also evaluated the same parameters in a control group consisting of 121 healthy children. The unexpected results of our study are presented below.

## 2. Material and Methods

### 2.1. Study Participants

The study included patients from two centers in Poland running the HPN program. The study group included 83 pediatric patients from all over Poland (31 females and 52 males) aged from 7.3 months to 18 years on long-term TPN conducted between 2004 and 2022. In 40 patients, PN was started in the neonatal period—group 1, and in 43 patients, PN was started over one month of age—group 2 (Table 1). The duration of PN measured up to the date of collection of the study material ranged from 4.6 months to 16.75 years. The reasons for implementing PN are shown in Table 2.

Inclusion criteria were individualized, long-term (≥1 month) TPN, and patient age from 2 months to 18 years at the time of investigation. The exclusion criterion was renal failure requiring dialysis. The supporting information can be found in Table S1 (https://docs.google.com/spreadsheets/d/1-hzeaFzOYCWR45F3vKZnEnU2MfR8kUC4/edit?usp=drive_link&ouid=118126989599175476191&rtpof=true&sd=true).

The mixtures for PN were prepared in the nutrition labs of hospital pharmacies. All study patients received more than 50% of energy from PN nutrition. All-in-one mixtures containing amino acids, glucose, lipid emulsion, electrolytes, vitamins, and trace elements were prepared according to the patient’s needs depending on age, weight, and metabolic status resulting from the underlying disease. 

In the study group, serum Al levels were assessed in all 83 patients, and urinary Al excretion in relation to urinary creatinine levels was assessed in 74 patients. 

The control group consisted of 121 healthy, born at term children (54 females and 67 males) aged from 1 month to 18 years from northwestern Poland. To evaluate the health status of children in the control group, a comprehensive physical examination was conducted, including an assessment of key anthropometric parameters such as height and body weight. The results of the measurements were related to the Polish centile grids appropriate for gender (https://www.ptzkd.org/new/standardy-i-zalecenia/ URL accessed on 12 August 2023). Inclusion criteria were the absence of chronic and acute diseases, weight, and height parameters within normal limits for age and sex.

The study group and the control group were similar in terms of age. None of the patients in both groups exhibited clinical symptoms of toxic Al effects.

In the control group, serum Al concentration was assessed in 121 children, and urinary Al excretion in relation to urinary creatinine concentration was assessed in 114 subjects. 

The samples of serum and urine were gathered from both the study and control groups during the years 2021 and 2022. The length of time that the samples were stored, measured in months, was compared between the study group (with a median of 14 months, a minimum of 6 months, a maximum of 22 months, and an interquartile range of 6) and the control group (with a median of 10 months, a minimum of 9 months, a maximum of 22 months, and an interquartile range of 4). These storage times were not found to be statistically different with a *p*-value of 0.52.

### 2.2. Sample Tubes

In order to ensure the highest level of accuracy, various types of blood collection tubes were examined to determine background elemental concentrations, with a particular focus on Al levels. Among the different manufacturers evaluated, including Sarstedt and Becton Dickinson, only one tube proved suitable for determining Al levels in patients’ samples. The BD Vacutainer #454001 tube was selected for collecting urine and blood samples for serum preparation due to its exceptionally low Al levels (<0.1 μg/L). To minimize any potential contamination, all tubes used in the dilution process were thoroughly rinsed twice with deionized water.

### 2.3. Trace Element Determination

The blood samples for serum were obtained from fasting individuals through venipuncture using the Vacutainer^®^ System (BD, EST Z #362725, Plymouth, UK). After collection, the tubes were allowed to clot at room temperature for a minimum of 30 min. Subsequently, the tubes were centrifuged at 1300× *g* for 12 min. Following centrifugation, the serum was carefully divided into new cryovials and then frozen at −80 °C until analysis. Similarly, urine samples were aliquoted into new cryovials immediately after collection and also stored at −80 °C until analysis. Prior to analysis, the urine samples were centrifuged at 5000× *g* for 5 min.

The elemental composition of the samples was determined using the inductively coupled plasma mass spectrometry (ICP-MS) technique with the NexION 350D instrument (PerkinElmer, Norfolk, VA, USA). The KED (Kinetic Energy Discrimination) mode was employed for element determination, and rhodium was used as an internal standard to compensate for instrument drift and matrix effects. Detailed information regarding the specific parameters of the NexION 350D instrument used in the measurements can be provided upon request. During analysis, the serum samples were diluted 30-fold with blank reagent, while the urine samples were diluted 10-fold with blank reagent.

The blank reagent used consisted of high-purity water (>18 MΩ), TMAH (AlfaAesar, Kandel, Germany), Triton X-100 (PerkinElmer), nitric acid (Merck, Darmstadt, Germany), and ethyl alcohol (Merck).

Calibration curve standards were prepared by diluting the stock solution of 10 mg/L Multi-element Calibration Standard 3 (PerkinElmer Pure Plus, Shelton, CT, USA) with the blank reagent. The calibration method used was matrix matched, and the correlation coefficients for each calibration curve were always greater than 0.999.

Aluminum excretion was assessed by calculating the aluminum/creatinine (Al/Cre) ratio.

### 2.4. Quality Control

The accuracy and precision of the measurements were evaluated using certified reference materials (CRM): ClinChek^®^ Plasmonorm Serum Trace Elements Level 1 (Recipe, Munich, Germany) for serum samples and ClinChek^®^ Urine Control Level 1 (Recipe, Germany) for urine samples. Technical details, plasma operating settings, and mass spectrometer acquisition parameters can be provided upon request. The testing laboratory participates in two independent external quality assessment schemes: LAMP (Lead and Multielement Proficiency Program) organized by the CDC (Center for Disease Control), and QMEQAS (Quebec Multielement External Quality Assessment Scheme) organized by the Institut National de Santé Publique du Québec.

### 2.5. Statistical Analysis

The Shapiro–Wilk test was used to assess the normality of the data distribution, and it was determined that the data did not follow a normal distribution. Descriptive statistics such as the median, interquartile range, mean, and standard deviation were used to summarize the analysis results. The Mann–Whitney U test was employed for data analysis. The correlation between variables was assessed using Spearman’s rank order test. A significance level of *p* < 0.05 was established for determining statistical significance.

## 3. Results

Serum Al concentration was determined in all 83 patients in the study group and 121 patients in the control group. Urinary Al excretion was determined in 74 patients in the study group and 114 patients in the control group. 

Table 3 shows the results of serum Al levels, urinary Al excretion, and the duration of PN in the study group, divided into patients in whom TPN started in the neonatal period (group 1) and patients in whom TPN started after one month of age (group 2).

There were no statistically significant differences in the duration of PN between children who started PN in the neonatal period and patients who started PN at more than one month of age.

Table 4 shows the results of serum Al levels and urinary Al excretion in the study group of patients divided into premature babies (<38 weeks gestational age) and babies born at term (≥38 weeks gestational age).

Table 5 shows the results of assessing serum Al levels and urinary Al excretion in the study group and the control group and the age characteristics of these groups.

## 4. Discussion

In 2022, Coulson and Hughes conducted a comprehensive review of the literature spanning from early 1966 to late 2020, focusing on the dose-dependent toxicity of aluminum (Al) in humans [29]. It shed light on the scarcity of studies that have measured Al concentrations in serum, plasma, blood, or urine, particularly in children.

Our study represents a significant contribution as it is the first to determine serum and urine Al concentrations in a large cohort of patients receiving long-term, home-based PN. Additionally, we present the Al concentration results in the serum and urine of a control group comprising healthy children.

According to Yokel [3], the administration of Al intravenously poses the greatest risk of the accumulation of this element due to its complete bioavailability and its strong binding affinity to transferrin. Consequently, patients, especially pediatric patients, who receive PN contaminated with Al from the formulations used to prepare nutritional mixtures, are at the highest risk of experiencing toxic effects from this element. Among this patient group, neonates, particularly premature infants, are exceptionally vulnerable due to the immaturity of their kidneys, which are responsible for eliminating Al [3,5,18,28]. Neonates retain approximately 75% of the parenterally administered Al, while adults retain 40% [2], and this Al deposition in tissues persists for many years, with an estimated half-life of 7 years [30].

Numerous studies have identified premature infants as the most susceptible group to Al toxicity. Early reports by Koo et al. in 1986 [25] and Stockhausen et al. in 1990 [31] highlighted the increased skeletal stress in infants receiving PN. A pivotal study conducted by Bishop et al., involving 227 premature infants receiving short-term PN (5–16 days), compared the neurological development of patients receiving standard PN solutions with those receiving Al-reduced PN solutions. The study revealed a reduction in the Bayley Mental Developmental Index score by 1 point for each day of feeding [9]. A follow-up study conducted by Fewtrell et al. 15 years later demonstrated reduced bone mass during adolescence [10]. These studies played a crucial role in the establishment of FDA regulations governing Al content in PN mixtures [14].

Our study reveals a clear association between the age of introduction of PN and serum Al concentration and urinary excretion in the study group of patients. We observed that children who initiated PN in the neonatal period had significantly higher serum Al concentrations and urinary excretion compared to patients who started PN at more than one month of age (*p* < 0.03). The median serum Al concentration in children who began PN in the neonatal period was 10.7 μg/L (range 4.46–46.07), and the median urine concentration was 163.8 μg/g creatinine (range 0.84–1282.09). Notably, the duration of PN until the day of study collection did not differ statistically between the two patient groups. These findings confirm previous evidence that the highest accumulation of Al occurs during the neonatal period in patients receiving parenteral feeding [3,5,18].

We did not observe statistically significant differences in serum Al levels and urinary excretion between preterm and term-born babies within the study group. This aligns with a study by Moreno et al., which demonstrated the risk of Al overload in not only preterm infants, but also term-born newborns, based on Al content in tissues and monitoring Al excretion in [26]. Moreover, our study revealed that the median serum Al concentration in the entire study group was 8.12 μg/L (range 1.54–47.7), exceeding the maximum concentration recommended by the German Federal Environmental Agency for the general population (<5 μg/L) [16]. Among the 83 patients in the study group, only 10 (12%) exhibited Al concentrations below 5 μg/L. The median urinary Al excretion in the study group was 83.61 μg/g creatinine (range 0.84–2762.18), surpassing the recommendation set by the German Research Foundation (50 μg Al/g creatinine) [16].

In 1986, Koo et al. conducted a study examining serum and urine Al concentrations in 20 infants who were fed parenterally for an average of 43 days. The infants were divided into two groups based on low and higher Al loads. The study found no difference in serum Al concentration based on exposure, but the median serum Al concentration was 37 μg/L. Urinary Al concentrations were higher in the group with higher Al loads, but there was no significant difference between newborns born at term and preterm infants [25]. 

The nearly five-fold higher median serum Al concentration observed in the infants from the 1986 study may be compared with the lower Al exposure in our study group, where serum Al concentrations exceeding 37 μg/L were found in only two patients. 

Assessing the total exposure to Al in our study group was not possible due to the long-term feeding period (up to 16.75 years) spanning from 2004 to 2022. Various formulations available during this time were used to create PN mixtures, and many changes were made in accordance with FDA recommendations to limit Al supply in PN. Over the years, numerous studies have evaluated the Al content in LVPs and SVPs [5,11,18,20,23,24,32].

A 2009 study by Popinska et al., conducted at one of the centers from which a significant proportion of our study group patients came, assessed Al concentrations in 24 patients on long-term PN. The study compared the Al concentration in the additives used for PN mixtures. In all subjects, the Al supply exceeded FDA recommendations, with younger patients having a low body weight being the most burdened due to higher calcium requirements compared to older children [24]. A study by Huston et al. published in 2017 showed that certain solutions, such as calcium gluconate in plastic packets, calcium chloride, sodium phosphate, and sodium glycerophosphate, have low Al content and were recommended by the authors for use in neonatal PN mixtures [20].

In a 2018 analysis by Emre et al. of the Al content in the LVPs and SVPs available in Turkey, as well as the mixtures made for newborns, it was found that the rate of Al exposure in preterm infants on TPN was much higher than the FDA-recommended safe dose [33].

In our study, the serum Al concentration in the control group (median 55.42 μg/L, range 6.13–413.86) was surprisingly almost seven times higher than that in the study group (median 8.12 μg/L, range 1.54–47.7) and more than ten times higher than the reference values for serum Al concentration (<5 μg/L) set by the German Federal Environmental Agency for the general population [16]. At the same time, urinary Al excretion in the control group is significantly lower than in the study group (*p* < 0.001). It is essential to consider the reasons for the high Al concentrations in the blood serum of healthy children and why no symptoms related to Al toxicity are found in them. 

Aluminum is naturally present in the environment and can enter the human body through various routes, including ingestion, inhalation, and skin absorption. Drinking water is a significant source of Al, with an average exposure of about 70 μg/L [3]. It is also commonly used in processed foods as additives, such as baking powder, cheeses, flavors, anticaking agents, spices, and beverages. Aluminum can be found in most food items, including meat, fish, grain products, and dairy products. Certain plants, such as tea leaves, have a tendency to accumulate Al, with raw tea containing approximately 600 mg/kg of the element [2,34].

Beverages, depending on regional preferences, can be a major source of Al intake. Additionally, the use of aluminum utensils and packaging significantly increases the Al content in food products [2,16,35]. It is worth noting that infant formula compositions can vary, and Al content in infant formulas ranged from 6 μg/L to 1152 μg/L in a study by Dabeka et al., with higher concentrations observed in soy, lactose-free, and hypoallergenic formulas [36]. The daily dietary exposure to Al from food is estimated to be between 5000 and 10,000 μg, according to Pennington and Schoen [37]. Antacids and phosphate antacids can also be significant sources of aluminum, with the adult intake reaching up to 3500–5200 mg/day [35,38]. Aluminum is also used as an adjuvant in vaccines and can be found in cosmetic products such as antiperspirants, toothpaste, and sun creams [3,16,35]. 

Inhalation exposure to Al primarily occurs in occupational and environmental settings. Studies by Kiesswetter et al. and Buchta et al. have shown that “Al content in welding fumes correlates with concentrations found in blood and urine, and subclinical changes associated with Al toxicity were observed in welders at a median serum Al concentration of 120 μg/L and urinary excretion of 100 μg/g creatinine” [39,40]. The daily exposure to Al through inhaled air is estimated to be less than 60 μg/day, according to Krewski et al. [41].

The bioavailability of Al and its health effects are influenced by factors such as the route, duration, and dose of exposure. Numerous studies have indicated that Al is poorly absorbed through various exposure routes, including oral ingestion, inhalation, transdermal absorption, and intramuscular or subcutaneous routes [3,16,42]. According to the Agency for Toxic Substances and Disease Registry (ATSDR) (2008), the absorption of Al through inhalation ranges from about 1.5 to 2%, while gastrointestinal absorption ranges from 0.01 to 5% and depends on factors such as the chemical form of Al, particle size, and exposure to chelators including citric acid and lactic acid [42]. The absorption of Al from the lungs appears to be more efficient compared to absorption from the gastrointestinal tract [43].

The brief literature review presented above highlights the widespread presence of Al. Considering its dose-dependent bioavailability, the elevated serum Al concentrations observed in the control group of children could potentially be explained by the high exposure to this element in their region of residence. It would be necessary to investigate the Al content in water sources and analyze the dietary habits of the children included in the study. In the case of infants, assessing the Al content in the modified mixtures they consume would also be important. The excessive intake of Al can surpass the limits of total fecal excretion, leading to the absorption and systemic circulation of Al despite the protective barrier of the gastrointestinal tract [11]. Furthermore, given the effective absorption of Al from the lungs, air pollution should be considered as a potential factor. Notably, within a radius of approximately 30 km, chemical plants produce nitrogen fertilizers and pigments treated with Al compounds on the surface. Additionally, greater control over the supply of antacids in children should be taken into account.

A nationwide study would be required to assess the serum Al concentrations in healthy children objectively. The control group in our study represents only a specific region in northwestern Poland, so we cannot directly compare its results with those of the study group consisting of children on TPN residing throughout Poland. However, we present this control group due to the unexpectedly high serum Al concentrations found in children who showed no clinical symptoms of toxic Al effects. It would be interesting to conduct neuropsychological testing in this group. However, considering the study by Kiesswetter et al. (2007) and Buchta et al. [39,40], it was observed that the first subclinical changes in adults occurred only at a median serum Al concentration of 120 μg/L.

## 5. Conclusions

Our study found that children receiving long-term PN experience significant exposure to aluminum, as evidenced by the elevated levels of this element found in serum and urine. Only 12% of the children studied on chronic PN displayed serum Al concentrations of less than 5 μg/L. 

There was a clear association between the age of PN introduction and serum Al concentrations and urinary excretion in the study group. Starting PN in the neonatal period increased serum Al concentrations and urinary excretion, although preterm labor did not affect these parameters. 

Contrary to expectations, healthy children in the control group had higher serum Al concentrations than those in the parenteral nutrition group. The influence of diet and environment may explain this observation, but it requires a large population-based study.

## Figures and Tables

**Table 1:** Characteristics of study group.

Parameter	Number of Patients Group 1	Number of Patients Group 2
Gestational age < 38 weeks	29	21
Gestational age ≥ 38 weeks	11	22
Birth weight > 2500 g	20	34
Birth weight ≤ 2500 g	20	9
Girls	17	14
Boys	23	29

**Table 2:** Reasons for implementing PN in the study group.

Underlying Disease	Number of Patients
Congenital mesenteric torsion	11
Hirschprung’s disease	14
Microvessel inclusion disease	1
Intestinal atresia	4
Expectoration	7
Torsion of the small intestine	5
Berdon syndrome	2
Pagoda syndrome	4
Neurogenic bowel	6
Absorption disorders	4
Uncompleted intestinal diversion	3
Necrotizing enterocolitis	12
Other intestinal malformations	10

**Table 3:** Al concentration in serum, urinary Al excretion, and duration of PN in the study group, divided into group 1 and group 2.

	Al in Serum [μg/L]		Al in Urine [μg/g Creatinine]		Duration of PN [Months]	
	Group 1	Group 2	Group 1	Group 2	Group 1	Group 2
Group size	40	43	36	38	40	43
Median	10.7	6.7	163.8	57.15	88.5	63.0
Range [Min–Max]	4.64–46.07	1.54–47.7	0.84–1282.09	0.85–2762.18	7.0–201.0	4.6–202.0
Interquartile range [IQR]	7.63	4.34	266.0	147.33	95.75	97.75
*p*-value	*p* < 0.03		*p* < 0.03		*p* > 0.05	

Serum Al concentration and urinary Al excretion in children who started PN in the neonatal period was significantly higher than in patients who started PN at more than one month of age (*p* < 0.03).

**Table 4:** Al concentration in serum and urinary Al excretion in the study group divided into preterm and term-born babies.

	Al in Serum [μg/L]		Al in Urine [μg/g Creatinine]	
	Premature Babies	Babies Born at Term	Premature Babies	Babies Born at Term
Group size	50	33	45	29
Median	9.17	6.98	116.43	60.78
Range [Min–Max]	3.3–46.07	1.54–47.7	0.84–1282.09	15.27–2762.18
Interquartile range [IQR]	6.35	6.5	223.16	150.32
*p*-value	*p* > 0.05		*p* > 0.05	

There were no statistically significant differences in serum Al levels and urinary Al excretion between preterm and term-born babies in the study group.

**Table 5:** Serum Al concentration and urinary Al excretion in the study group and the control group and age characteristics.

	Al in Serum [μg/L]		Al in Urine [μg/g Creatinine]		Age [Months]	
	Study Group	Control Group	Study Group	Control Group	Study Group	Control Group
Group size	83	121	74	114	83	121
Median	8.12	55.42	83.61	12.71	93.93	95.0
Range [Min–Max]	1.54–47.7	6.13–413.86	0.84–2762.18	1.6–193.1	7.27–219.3	1.0–216.0
Interquartile range [IQR]	6.47	28.16	216.73	20.35	121.93	93.3
*p*-value	*p* < 0.001		*p* < 0.001		*p* > 0.05	

Serum Al concentration was significantly higher in the control group than in the study group (*p* < 0.001). Urinary Al excretion was significantly lower in the control group than in the study group (*p* < 0.001). The age of patients in the study group and the control group was not statistically different.

## Data Availability

The data presented in this study are available upon request from H.R.

## Supplementary Materials

The following supporting information can be downloaded at: https://docs.google.com/spreadsheets/d/1-hzeaFzOYCWR45F3vKZnEnU2MfR8kUC4/edit?usp=drive_link&ouid=118126989599175476191&rtpof=true&sd=true, Table S1: Database—study group and control group.
